# Differential gene expression is not required for facultative sex allocation: a transcriptome analysis of brain tissue in the parasitoid wasp *Nasonia vitripennis*

**DOI:** 10.1098/rsos.171718

**Published:** 2018-02-21

**Authors:** Nicola Cook, Rebecca A. Boulton, Jade Green, Urmi Trivedi, Eran Tauber, Bart A. Pannebakker, Michael G. Ritchie, David M. Shuker

**Affiliations:** 1 School of Biology, University of St Andrews, Greenside Place, St Andrews KY16 9TH, UK; 2 Department of Entomology, University of Minnesota, St Paul, MN 55108, USA; 3 Edinburgh Genomics, University of Edinburgh, Ashworth Laboratories, The King's Buildings, Edinburgh EH9 3FL, UK; 4 Faculty of Natural Sciences, University of Haifa, 199 Aba Khoushy Avenue, Mount Carmel, Haifa 3498838, Israel; 5 Laboratory of Genetics, Wageningen University and Research, Droevendaalsesteeg 1, 6708PB Wageningen, The Netherlands

**Keywords:** sex allocation, behavioural genetics, transcriptomics, parasitoid, local mate competition, *Nasonia*

## Abstract

Whole-transcriptome technologies have been widely used in behavioural genetics to identify genes associated with the performance of a behaviour and provide clues to its mechanistic basis. Here, we consider the genetic basis of sex allocation behaviour in the parasitoid wasp *Nasonia vitripennis*. Female *Nasonia* facultatively vary their offspring sex ratio in line with Hamilton's theory of local mate competition (LMC). A single female or ‘foundress’ laying eggs on a patch will lay just enough sons to fertilize her daughters. As the number of ‘foundresses’ laying eggs on a patch increases (and LMC declines), females produce increasingly male-biased sex ratios. Phenotypic studies have revealed the cues females use to estimate the level of LMC their sons will experience, but our understanding of the genetics underlying sex allocation is limited. Here, we exposed females to three foundress number conditions, i.e. three LMC conditions, and allowed them to oviposit. mRNA was extracted from only the heads of these females to target the brain tissue. The subsequent RNA-seq experiment confirmed that differential gene expression is not associated with the response to sex allocation cues and that we must instead turn to the underlying neuroscience to reveal the underpinnings of this impressive behavioural plasticity.

## 1. Introduction

The genomics revolution has brought several new technologies to behaviour genetics. For instance, the top-down assessment of genetic variation underlying quantitative traits can now be extended through genome-wide association studies using large-scale single nucleotide polymorphism datasets [1,2]. In terms of bottom-up approaches, gene knockdown and gene editing techniques, such as RNAi [3–5] and CRISPR-cas9 [6,7], respectively, have also promised to help unpick molecular mechanisms underpinning behaviour on a gene by gene, or nucleotide by nucleotide basis. However, it has been the development of accessible whole-transcriptome technologies (from microarrays to RNA-seq) that have perhaps been the most widely used, not least because they help fill the gap between top-down and bottom-up approaches to behaviour genetics [8], and because they can be applied in non-model species [9].

Transcriptome studies can approach the genetic basis of behaviour in two ways. First, surveys of expressed genes can identify what genes and gene networks are associated with the performance of a behaviour. Recent examples in non-model insects include parental care in the burying beetle *Nicrophorus vespilloides* [10] and oviposition behaviour in the parasitoid wasp *Nasonia vitripennis* (Walker, 1836) [11]. However, a great many genes may be expressed during the action of a behaviour (especially over the time scale usually assayed, i.e. at least minutes, if not hours) many of which will have little or no direct relationship to facilitating the performance of that behaviour. The most obvious example would be genes that are upregulated or downregulated as a consequence of a particular behaviour, rather than genes that are causal [12]. While the former is undoubtedly useful for expanding our understanding of the genetic, and perhaps physiological, context of behaviour, it is less helpful in identifying the genes that bring a behaviour into being.

Second, transcriptomic studies of behaviour often involve identifying the changes in gene expression that occur when a behaviour commences or when a behaviour shifts. For example, the transition between virgin and ‘mated’ reproductive status in honeybee queens is associated with significant changes in the expression of chemo-reception, metabolomic, vision and immune-related genes [13]. However, the time scale of analysis again makes it difficult to separate behavioural cause and consequence of the mating process, i.e. which expression changes are attributable to the performance of, for example, the mating flight and/or copulation and which are a consequence of those behaviours having occurred. Nevertheless, experimental manipulation of behaviour has helped identify genes and gene networks that appear to have a causal relationship with the performance of a behaviour, i.e. ‘behaviour genes’ [14].

Here, we consider the genetic basis of sex allocation in the parasitoid wasp *Nasonia vitripennis*. Female *Nasonia* facultatively vary their offspring sex ratios in line with Hamilton's theory of local mate competition (LMC) [15]. Females parasitize locally discrete patches of blowfly pupae, with emerging offspring mating before females disperse to found the next generation [16]. If only one female lays eggs on a patch, then on emergence, related males (i.e. brothers) compete for mates among their sisters. Hamilton showed that this favours mothers producing female-biased sex ratios that reduced competition among brothers and increased the number of sisters available for them to mate with [15,17]. Indeed, a single female laying eggs on a host—termed a ‘foundress’ in the parasitoid literature—should produce the minimum number of sons needed to fertilize her daughters. However, if multiple foundresses lay eggs together, the degree of competition among related males is reduced (in Hamilton's parlance, the degree of local mate competition is reduced), and so less female-biased sex ratios are favoured. One way to conceptualize this reduction of LMC as more females lay eggs together is that as the number of foundress females increases, the fitness returns of sons and daughters becomes more equal, and a mother is as successful at producing grand-offspring through sons as through daughters.

To allocate sex in line with LMC predictions, female *Nasonia* need to estimate the extent to which their sons will face kin versus non-kin in terms of mate competition, i.e. the extent of LMC. Since the pioneering work of Werren [18–20], we have collected an impressive array of phenotypic studies that have confirmed that female *Nasonia* pay attention to a whole range of LMC cues [21–25]. Our understanding of the genetics underlying this impressive behavioural plasticity is much more limited however. Early work by Orzack and co-workers confirmed the presence of genetic variation in sex allocation in *N. vitripennis* [26], and more recent work has measured the mutational heritability of single-foundress sex ratios [27] and also identified quantitative trait loci associated with sex ratio variation [28]. To move beyond these quantitative genetic approaches, Pannebakker *et al.* [11] used a whole-transcriptome approach to explore changes in gene expression associated with oviposition (a necessary part of sex allocation, as eggs are fertilized or not immediately prior to oviposition: as a haplodiploid insect, fertilized diploid eggs develop into females, and unfertilized haploid eggs develop into males [29]). They showed that 332 genes displayed different expression patterns when females were ovipositing when compared with resting controls, with the majority of the changes associated with the downregulation of genes associated with metabolism.

Most recently, Cook *et al*. [30] extended this study to compare patterns of gene expression associated with facultative sex allocation, with transcriptomes screened in females that were either alone or in groups of 10 (‘social’ LMC cues [31]), and that were either given no hosts, unparasitized hosts, or already parasitized hosts (‘host’ LMC cues), in a fully factorial 3 × 2 design. From the phenotypic studies, we knew that females should produce different sex ratios when ovipositing in the presence of other co-foundresses and/or when ovipositing on hosts that had already been parasitized (the so-called superparasitism [32]). However, there was no evidence of changes in gene expression associated with co-foundresses or superparasitism. Instead, there was a strong pattern of differential gene expression (DGE) associated with the presence or the absence of hosts (i.e. whether females were ovipositing or not), with 1359 genes showing significant DGE. This study therefore confirmed that oviposition leads to changes in gene expression in female *Nasonia*, but strongly suggested that facultative sex allocation does not.

Both Cook *et al.* [30] and Pannebakker *et al.* [11] harvested mRNA from whole bodies. While this is still common for behavioural transcriptome studies, especially in non-model insects, whole-body approaches have been criticized; selecting only the tissues most relevant to the behaviour will increase the relevance of the gene expression data [12]. To begin to address this, here we performed an RNA-seq experiment using mRNA harvested from the heads of female *N*. *vitripennis*, to target the brain tissue of the wasps. While the neural basis of oviposition and sex allocation behaviour is unknown (but see below), it is likely that regions of the brain such as the mushroom bodies are sites of information processing that should be relevant for such behavioural decisions [33]. We allowed females to lay eggs in one of three foundress conditions (alone, with four co-foundresses or with nine co-foundresses) and tested whether the facultative sex allocation in response to foundress number is associated with DGE across the *N. vitripennis* transcriptome.

## 2. Material and methods

### 2.1. Study species

*Nasonia vitripennis* (Hymenoptera, Chalcidoidea) is a generalist parasitoid of large dipteran pupae including species of Calliphoridae. Females oviposit between 20 and 50 eggs in an individual host, with male offspring emerging just before females (after approx. 14 days at 25°C [16]). Males are brachypterous and unable to fly, remaining close to the emergence site where they compete with each other for emerging females, including their sisters. Females disperse after mating to locate new hosts. The focal females used in this experiment were from the AsymC strain, originally isolated in 1986 by curing the wild-type strain LabII of *Wolbachia* and is known to be free of sex-ratio distorters [34,35]. Wasps have been maintained on *Calliphora vomitoria* or *C. vicina* hosts at 25°C, 16 L : 8 D light conditions ever since. Where co-foundresses were required, these were taken from the red-eye mutant STDR strain, allowing us to track the offspring of a single AsymC female using eye colour. The STDR strain is maintained under conditions identical to the AsymC strain.

### 2.2. Experimental design

To control for possible host and other maternal effects, experimental females were not drawn straight from stock populations. Instead, 2-day-old, mated, wild-type AsymC females were isolated from the mass cultures into individual glass vials. Each female was provided with three hosts and allowed to oviposit. Experimental females were drawn from the resulting F_1_ generation, one female per ‘grandmother’. We balanced the emergence of the F_1_ generation over three days, and therefore the experimental set-up, so that we needed to process fewer replicates simultaneously. This reduced the time-difference between the harvesting of females for RNA extraction (see below).

Experimental females were pre-treated in the first instance by provision with a single host for 24 h and then provision with honey solution for the following 24 h. This pre-treatment procedure allows host-feeding and facilitates egg development. We then employed a simple three-treatment experimental design, with *N* = 135 replicates per treatment (total *N* = 405 experimental females), balanced over 3 days. Females were allocated to one of three ‘foundress number’ groups: (i) single foundress, (ii) five foundress (i.e. one experimental female plus four STDR co-foundresses ovipositing simultaneously) or (iii) 10 foundress (i.e. one experimental female plus nine STDR co-foundresses ovipositing simultaneously). Females were given access to three hosts and allowed to explore the hosts and oviposit for 3 h. At the end of the 3 h period, focal females were identified by eye colour and their heads excised and placed into RNAlater (ThermoFisher Scientific, Waltham, MA, USA) for storage at −20°C in advance of RNA extraction. Hosts were returned to the incubator and the emergent offspring counted and sexed to verify that the expected sex allocation response was noted in this experiment (electronic supplementary material, figure S1; females produced increasingly male-biased sex ratios with an increase in foundress number). Only the heads of females that produced offspring were used for RNA extraction. Heads were pooled into groups of 12 within treatment group (*n* = 8 pools per treatment, *N* = 24 overall) and stored at −20°C in advance of RNA extraction.

### 2.3. RNA extraction

RNA was isolated from 12 pooled heads using the TRIzol Plus RNA Purification Kit in conjunction with the PureLink RNA Mini Kit (Life Technologies, Paisley, UK) according to the manufacturer's instructions. Additional steps for ‘On-Column PureLink DNase Treatment During RNA Purification’ were followed. Concentration and integrity of RNA samples were checked using a Nanodrop spectrophotometer (Nanodrop Technologies, Wilmington, DE) and a bioanalyser system (Agilent Technologies, Santa Clara, CA), respectively. Total RNA obtained was (mean total RNA available for library preparation = 0.855 µg, s.d. = 0.297 µg) of good quality (260/280 ≥ 1.8, RIN values ≥ 8.0 for all samples).

### 2.4. Library preparation and sequencing

Library preparation and sequencing were carried out by Edinburgh Genomics. As in our previous work [30], mRNA library preparation for paired-end sequencing was carried out using the Illumina TruSeq RNA Sample Prep Kit (Illumina, San Diego, CA) following the Illumina TruSeq Sample Preparation v2 (Low Sample) protocol. Briefly, mRNA was purified from total RNA samples using oligo-dT-attached magnetic beads and fragmented using divalent cations at 94°C. First-strand cDNA synthesis was carried out using reverse transcriptase and random hexamer primers. Second-strand synthesis was carried out using RNA polymerase I and RNase H. Overhangs resulting from fragmentation were converted to blunt ends and 3′ ends were subsequently adenylated. Sanger indexing adapters were ligated to the fragments that were then purified and PCR-amplified to create the final cDNA libraries for sequencing. Library preparation was successful for all but one sample; a single replicate from the five-foundress treatment group. A total of 23 libraries were sequenced on the HiSeq2000 (125 bp paired-end) according to the manufacturer's instructions. Raw sequence reads are available from the Gene Expression Omnibus database at NCBI (Accession: GSE105796).

### 2.5. Mapping, filtering and annotation

Raw reads were filtered for quality (reads with an average Phred quality score of 30 or higher over the length of the read were retained) and adapter contamination using cutadapt (v1.8.3). Filtered, adapter-trimmed reads were subsequently aligned to the *N. vitripennis* genome (Assembly Nvit 2.1: NCBI Accession: GCF_000002325.3) using tophat2 (v. 2.0.14). Read counts per gene were obtained using HTSeq (v. 6.0.1) ‘union’ mode with ‘NCBI *Nasonia vitripennis* Annotation Release 101’.

In terms of the dataset, we obtained 2 332 938 466 trimmed reads across all 23 sequenced libraries with 95.14% of reads mapped to Nvit 2.1 overall (see the electronic supplementary material, table S1, for individual library mapping statistics). Owing to the ‘union’ mode applied to HTSeq, some of the reads that mapped ambiguously to a gene were removed (i.e. not counted) and the final dataset comprised 886 364 361 reads mapped to 14 131 genes. For 13 599 of these genes, at least one read was mapped.

### 2.6. Statistical analyses

In the first instance, the count data were explored using DEseq2 [36] in the R environment [37] to check for any samples that were clear outliers. To this end, a regularized log transformation was applied to the count data and a principal component plot generated based on the count data from the 500 genes displaying the highest variance across all samples. One replicate from the single-foundress treatment group was found to be a clear outlier and was removed from subsequent analyses (electronic supplementary material, figure S2). This sample was subjected to identical experimental procedures as its corresponding replicates and the resulting RNA of similar high quality prior to library preparation and sequencing. Notably, this sample had the lowest percentage of trimmed reads mapped to the reference genome (electronic supplementary material, table S1) suggestive of an anomaly at the sequencing stage. The removal of this outlier had no effect on the results in terms of the number of significantly differentially expressed genes (DEG).

After the removal of outliers, DESeq2 was employed to test for differential expression between pairs of treatment groups by fitting a generalized linear model (GLM) for each gene and subsequently to determine whether each model coefficient differs significantly from zero. The Wald test was used for significance testing and the resulting false discovery rate (FDR) *p*-values were adjusted for multiple comparisons using the Benjamini and Hochberg method [38]. Adjusted *p*-values less than 0.05 would be considered indicative of DEG. Subsequently, we analysed the results comparing gene expression across the three treatments fitting the factor ‘foundress number’ in a GLM framework using the negative binomial error distribution and a likelihood ratio test for significance.

## 3. Results

Facultative sex allocation behaviour in response to co-foundresses is not associated with any short-term gene expression changes in the head, as a proxy for the brain, of female *N. vitripennis*. This result was consistent for all pairwise comparisons between the three treatment groups across 13 599 genes: single- versus five-foundress (FDR *p*-values all *p* > 0.99), five versus 10-foundress (FDR *p*-values all *p* > 0.99) and single- versus 10-foundress (FDR *p*-values all *p* > 0.1 bar a single gene (cytochrome P450 314A1, NCBI Gene ID: 100115247) for which *p* = 0.074). An additional test for an overall effect of ‘foundress number’ on gene expression using a GLM approach confirmed this (FDR *p*-values all *p* > 0.1).

**Figure 1.**
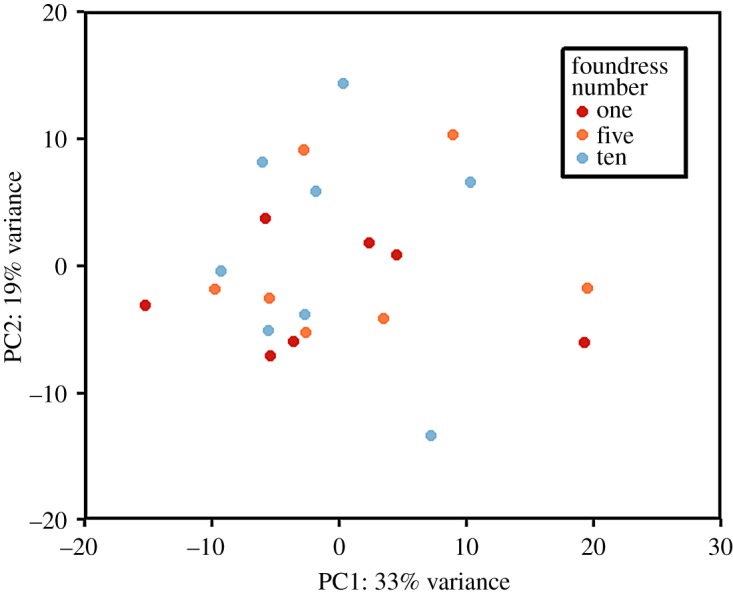
Principal component plot based on count data from the 500 genes displaying the greatest variance across all samples.

Prior to the application of adjustment for multiple comparisons, 215 genes were differentially expressed at *p* < 0.05 in the single- versus five-foundress comparison, 201 in the five- versus 10-foundress comparison and 427 in the single versus 10-foundress comparison. Similarly, 261 genes were differentially expressed at *p* < 0.05 in the factorial analysis prior to ‘false discovery rate’ correction. However, the lack of differentiation between treatment groups can be clearly seen in figure 1. Overall, these results strongly suggest that there is no differential gene expression associated with exposure to different sex allocation cues.

## 4. Discussion

The results presented here are in agreement with our previous findings [30] which showed that oviposition, i.e. the presence or the absence of a host, led to significant changes in gene expression at the level of the whole body in *N. vitripennis*, but that known sex allocation cues (the presence of co-foundresses or previously parasitized hosts) did not. Taken together, our results strongly suggest that the remarkable phenotypic plasticity that underlies facultative sex allocation in *Nasonia* does not involve systematic changes in gene expression.

That said, negative results bring their own evidential burden. While we are pleased to demonstrate that techniques used in our laboratory to assay differential gene expressions produce consistent results (especially given the recent concerns in the scientific community over reproducibility [39]), and while we also believe that we have a strong experimental design, we appreciate the risk of Type II and Type I errors. With any experiment, there is always a trade-off between cost and accuracy when selecting the number of biological replicates, and replication in RNA-seq studies has been a topic of discussion in the recent literature ([40–43] to name a few). In their comprehensive evaluation of 11 tools for RNA-seq data analysis, Shurch *et al.* [40] determined that at least six biological replicates should be used per treatment group and that with around this number of replicates Edge R [44] and DESeq2 [36] outperform other tools. Both of these tools are considered to have a superior true-positive identification rate and well-controlled FDR for genes exhibiting lower fold changes between treatment groups. The authors stated, however, that for *n* ≤ 12 biological replicates DESeq2 should be ‘the tool of choice’ and was therefore the most suitable for use in the current study.

To further illustrate the reliability of our results, we selected two replicates from each treatment group that showed ‘high divergence from others in their group’ in the principal component analysis plot (electronic supplementary material, figure S3) from the other two groups and ran an identical DESeq2 analysis to that presented here. This analysis, using only two biological replicates per treatment group, turned up 900 DEG when comparing single- versus five-foundress treatments, 49 DEG for five- versus 10-foundress treatments and 1397 DEG for single- versus 10-foundress treatments. Under a GLM framework, 1368 genes were differentially expressed in association with ‘foundress number’. This, firstly, highlights the importance of biological replication in RNA-seq studies as discussed by Shurch *et al.* [40] and, secondly, increases our confidence in the results presented here (i.e. by choosing the most divergent samples we can ‘find’ significant differential gene expression among our treatments, but such differences do not appear when using all the replicates). See Libbrecht *et al.* [45] for an analogous case with DNA methylation in social insects.

Behavioural transcriptomic studies on samples containing multiple tissue and/or cell types such as that reported here and previously (examples include [10,11,30,46]) have received criticism [47]. It has been suggested that non-isometric-scaling relationships, i.e. differences in size, within groups of samples and heterogeneity in scaling relationships across groups of samples may influence differential expression. This may result in false-positive or false-negative results that are attributable to non-isometric scaling at multiple biological levels rather than the trait in question [47]. However, this particular criticism is not applicable to our work, as the samples are comparable in size (either as whole bodies or as heads) and physiological state, i.e. there is no reason for any one treatment group to develop differently. As such, any issues with scaling should be negligible.

Another common criticism of whole-body transcriptomic studies is that the ‘signal’ of differential expression between treatment groups from some genes may be obscured simply because the regulation occurs in a single tissue or group of cells and the difference in expression is relatively low [9]. To try and evaluate this problem, in our previous work that only looked at whole bodies [30], we compared our list of 1359 genes that were differentially expressed during oviposition to a list of 79 genes and their associated peptides known to be both expressed and present in the venom gland and/or the ovary of *N. vitripennis* [48,49]. It is very likely that expression of these genes is highly concentrated in these female organs given that they function to envenomate the pupal host via the ovipositor prior to egg-laying. In spite of this, our original whole-body study picked up that 33 of these genes were differentially expressed in response to oviposition. Therefore, for two organs where DEG would seem extremely likely to be detected in response to oviposition, the signal was present even when sampling the whole insect.

We also considered whether the timing of our experiment may have prevented detection of DEG in response to sex allocation behaviour. To permit comparison with our previous work [30], we used the same timing: females were harvested after 3 h of exposure to hosts. In our previous experiment, we looked for DEG in response to both oviposition itself and sex allocation in a fully factorial design. We found clear evidence of DEG associated with oviposition but not with sex allocation [30]. It stands to reason that if we can detect DEG in association with oviposition at this time point, then we should also be able to detect any DEG associated with sex allocation; females allocate the sex of their offspring by releasing or withholding sperm to fertilize eggs or not as they are laid in order to allocate sex [16]. Thus, it seems that if DGE was involved in the process of sex allocation as distinct from oviposition, we would have been able to detect it using the methods described here.

Here, we have validated a negative result. We hypothesized that DGE in the neural circuitry, more specifically the brain, may be associated with the response to sex allocation cues but that it may not have been detected due to the whole-body approach. The results of the current study confirm that the response to sex allocation cues in female *N*. *vitripennis* is not facilitated by differential expression in the brain and so does not require or involve any significant alteration to gene regulation, while the process of oviposition itself does [30]. These findings, combined with our recent work on the effects of neonicotinoid pesticides—which disrupt synaptic nicotinic acetylcholine receptors—on sex allocation [50–52], mean that to understand the mechanistic basis of adaptive facultative sex allocation under LMC, we must instead now turn to the underlying neuroscience.

## Supplementary Material

Supplementary figures

## Supplementary Material

Supplementary tables

## Data accessibility

Raw sequence reads and processed data are available from the Gene Expression Omnibus hosted by NCBI (Accession: GSE105796).
