# Supplementary material for: Differential gene expression is not required for facultative sex allocation: a transcriptome analysis of brain tissue in the parasitoid wasp Nasonia vitripennis
Source: R Soc Open Sci. 2018 Feb 21;5(2):171718. doi: 10.1098/rsos.171718 (PMC5830769; doi:10.1098/rsos.171718)

**Figure S1:** Plot showing mean sex ratio produced in each of the “Foundress Number” treatment groups. Error bars are binomial confidence intervals. Number of females producing offspring and therefore used to calculate mean sex ratio is shown for each treatment.

**
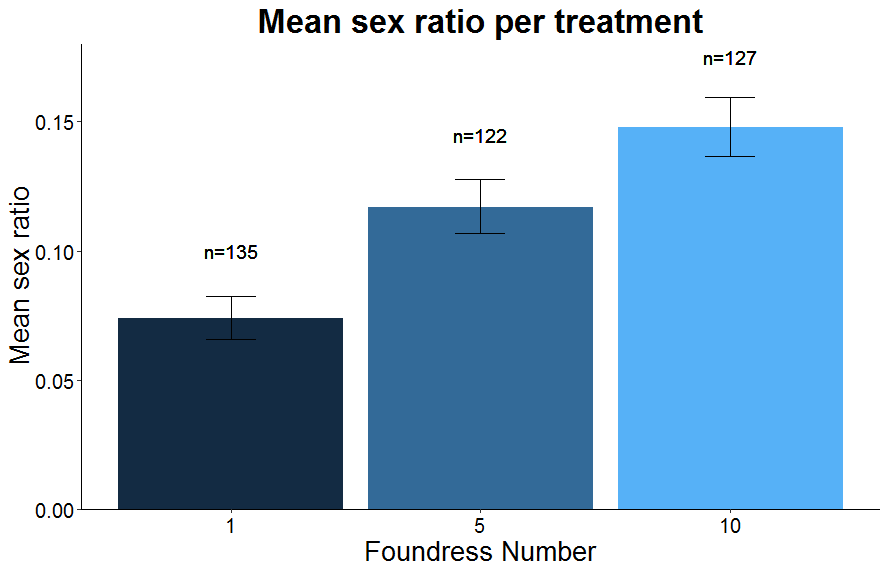
**

**Figure S2:** Principal Component Plots used to detect outliers**.** A) All 23 libraries included. B) After outlier Rep 2 from the single-foundress treatment group removed.

**A**


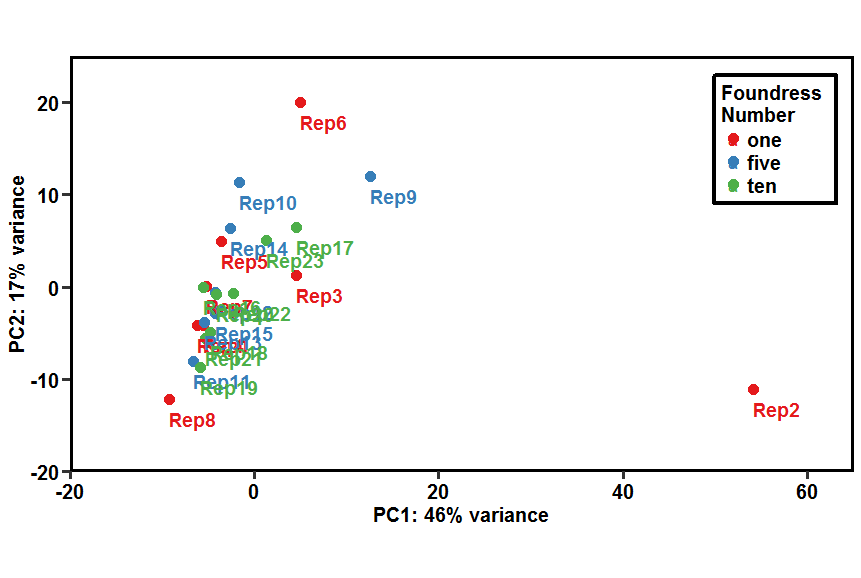


**B**

**
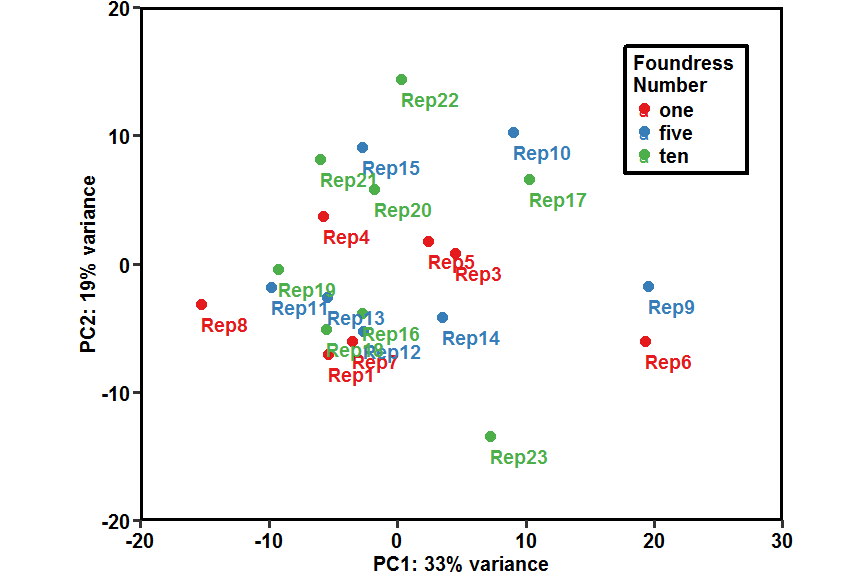
**

**Figure S3:** Principal Component Plot reproduced from Figure S1B showing replicates chosen to re-run the DESeq2 analysis (see Discussion).


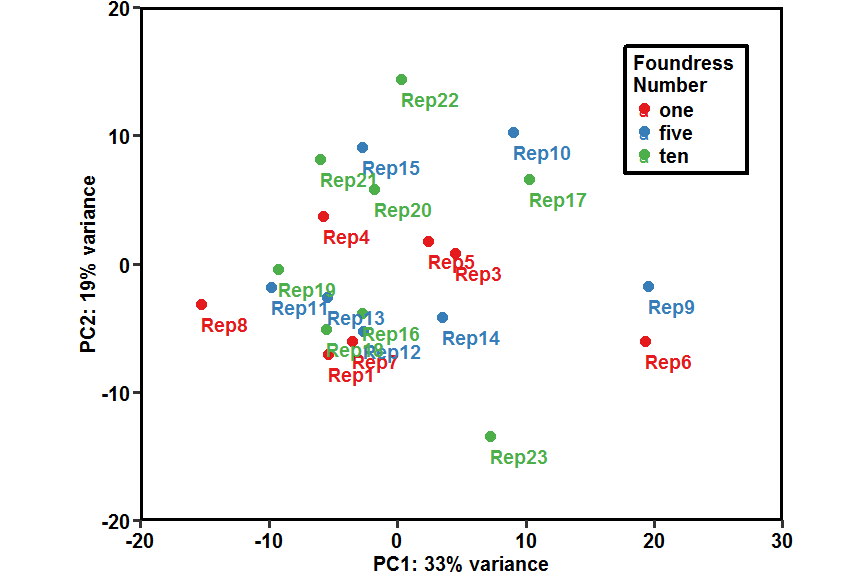

Supplement: Supplementary figures [file rsos171718supp1.docx]
