# Supplementary material for: Differential gene expression is not required for facultative sex allocation: a transcriptome analysis of brain tissue in the parasitoid wasp Nasonia vitripennis
Source: R Soc Open Sci. 2018 Feb 21;5(2):171718. doi: 10.1098/rsos.171718 (PMC5830769; doi:10.1098/rsos.171718)
Supplement: Supplementary tables [file rsos171718supp2.docx]

**Table S1:** Mapping statistics for all sequenced libraries

| **Sample Number** | **Treatment group** | **No. trimmed reads** | **No. mapped reads** | **Percentage of trimmed reads mapped** |
| --- | --- | --- | --- | --- |
| 1_FN1 | Single foundress | 88770560 | 84604957 | 95.31 |
| 2_FN1 | Single foundress | 137143190 | 126233772 | 92.05 |
| 3_FN1 | Single foundress | 98802842 | 94312445 | 95.46 |
| 4_FN1 | Single foundress | 88438900 | 84944334 | 96.05 |
| 5_FN1 | Single foundress | 119720666 | 113306434 | 94.64 |
| 6_FN1 | Single foundress | 75620908 | 72291937 | 95.60 |
| 7_FN1 | Single foundress | 74324786 | 70976954 | 95.50 |
| 8_FN1 | Single foundress | 81108364 | 76810796 | 94.70 |
| 10_FN5 | Five-foundress | 111481154 | 106624648 | 95.64 |
| 11_FN5 | Five-foundress | 142762414 | 136829209 | 95.84 |
| 12_FN5 | Five-foundress | 73391406 | 69827328 | 95.14 |
| 13_FN5 | Five-foundress | 93301620 | 89040381 | 95.43 |
| 14_FN5 | Five-foundress | 105697300 | 100718298 | 95.29 |
| 15_FN5 | Five-foundress | 66456262 | 63661685 | 95.79 |
| 16_FN5 | Five-foundress | 178449808 | 170921838 | 95.78 |
| 17_FN10 | Ten-foundress | 83090508 | 78751601 | 94.78 |
| 18_FN10 | Ten-foundress | 117939390 | 112718494 | 95.57 |
| 19_FN10 | Ten-foundress | 112639622 | 106974229 | 94.97 |
| 20_FN10 | Ten-foundress | 86295036 | 81103175 | 93.98 |
| 21_FN10 | Ten-foundress | 99262896 | 94709323 | 95.41 |
| 22_FN10 | Ten-foundress | 80433942 | 76007434 | 94.50 |
| 23_FN10 | Ten-foundress | 130260196 | 124908933 | 95.89 |
| 24_FN10 | Ten-foundress | 87546696 | 83381401 | 95.24 |
